# Inkjet drug printing onto contact lenses: Deposition optimisation and non-invasive dose verification

**DOI:** 10.1016/j.ijpx.2022.100150

**Published:** 2022-12-20

**Authors:** Thomas D. Pollard, Iria Seoane-Viaño, Jun Jie Ong, Patricija Januskaite, Sahar Awwad, Mine Orlu, Manuel F. Bande, Abdul W. Basit, Alvaro Goyanes

**Affiliations:** a Department of Pharmaceutics, UCL School of Pharmacy, University College London, 29-39 Brunswick Square, London WC1N 1AX, UK; b Department of Pharmacology, Pharmacy and Pharmaceutical Technology, Paraquasil Group (GI-2109), Faculty of Pharmacy, and Health Research Institute of Santiago de Compostela (IDIS), University of Santiago de Compostela (USC), Santiago de Compostela 15782, Spain; c Department of Ophthalmology, University Hospital of Santiago de Compostela, Ramon Baltar S/N, Santiago de Compostela 15706, Spain; d FabRx Ltd., Henwood House, Henwood, Ashford TN24 8DH, UK; e Departamento de Farmacología, Farmacia y Tecnología Farmacéutica, I+D Farma Group (GI-1645), Facultad de Farmacia, iMATUS and Health Research Institute of Santiago de Compostela (IDIS), Universidade de Santiago de Compostela (USC), Santiago de Compostela 15782, Spain

**Keywords:** Point-of-care dispensing, 2D printing, Personalized healthcare, Printing medicines, Process analytical technology (PAT) tools, Ophthalmic and ocular drug delivery, DMSO, dimethyl sulfoxide, HPLC, high performance liquid chromatography, NIR, near-infrared, PAT, process analytical technology, PBS, phosphate-buffered saline, PLS, partial least squares, RMSE, root mean square error, SCL, soft contact lens

## Abstract

Inkjet printing has the potential to advance the treatment of eye diseases by printing drugs on demand onto contact lenses for localised delivery and personalised dosing, while near-infrared (NIR) spectroscopy can further be used as a quality control method for quantifying the drug but has yet to be demonstrated with contact lenses. In this study, a glaucoma therapy drug, timolol maleate, was successfully printed onto contact lenses using a modified commercial inkjet printer. The drug-loaded ink prepared for the printer was designed to match the properties of commercial ink, whilst having maximal drug loading and avoiding ocular inflammation. This setup demonstrated personalised drug dosing by printing multiple passes. Light transmittance was found to be unaffected by drug loading on the contact lens. A novel dissolution model was built, and in vitro dissolution studies showed drug release over at least 3 h, significantly longer than eye drops. NIR was used as an external validation method to accurately quantify the drug dose. Overall, the combination of inkjet printing and NIR represent a novel method for point-of-care personalisation and quantification of drug-loaded contact lenses.

## Highlights

• Developed point-of-care personalised drug delivery system for ophthalmic diseases.
• Combined inkjet printing and NIR system for contact lens drug delivery.
• Inkjet printed drugs onto inside or outside face of contact lens.
• NIR showed good correlation as a quality control method.
• Developed novel drug dissolution apparatus and demonstrated extended release.

## Graphical abstract

**Figure:**
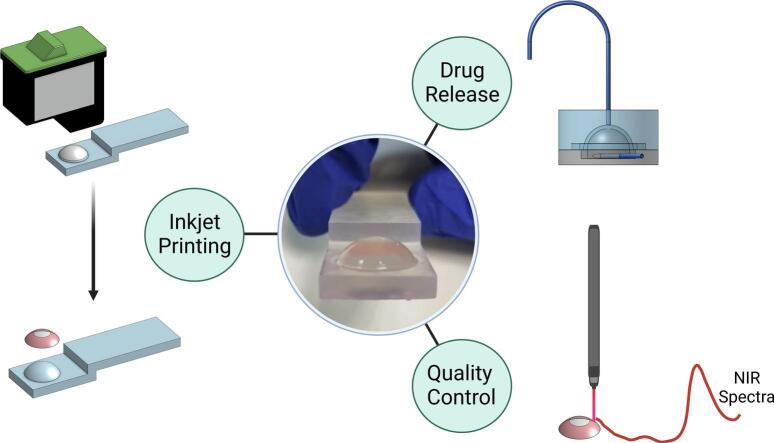

## 1 Introduction

Medicated eye drops are the current standard treatment for numerous common eye diseases, including glaucoma, fungal keratitis, and acute conjunctivitis. These ocular conditions affect people across socio-economic strata, with glaucoma being the leading cause of irreversible blindness worldwide and the number of diagnosed patients estimated to reach 111.8 million by 2040 (Allison et al., 2020; Kaur et al., 2022; Rossetti et al., 2016). However, topical delivery has been reported to result in poor bioavailability (<5%) due to various anatomical constraints such as the blood-aqueous barrier, blood-retinal barriers and the corneal epithelium, and physiological barriers to drug permeation, which include blinking and nasolacrimal drainage (Bachu et al., 2018; Patel et al., 2013). Furthermore, non-compliance to eye drop treatment is common, largely due to forgetfulness (Lacey et al., 2009; Waterman et al., 2013), difficulties with the medication schedule (Tsai et al., 2003), or difficulty in administering the eye drops (Stryker et al., 2010; Waterman et al., 2013). A study investigating glaucoma patients reported that 9 out of 10 individuals were unable to correctly administer eye drops (Gupta et al., 2012). Additionally, when contact lenses are worn, they must be removed before administering eye drops and not replaced for 15 min (FDA, 2013), which further contributes to patient inconvenience.

Drug-loaded soft contact lenses (SCLs) are an attractive form of ophthalmic drug delivery in pharmaceutical research (Pereira-da-Mota et al., 2022; Yang and Lockwood, 2022) due to the potential for sustained release, improved patient compliance, increased bioavailability, and a reduction in the dose necessary to reach a therapeutic effect. Different methods for incorporating drugs into SCLs exist (Akbari et al., 2021; Ciolino et al., 2016; Datta et al., 2022; Franco and De Marco, 2021; Hewitt et al., 2020; Hsu et al., 2015; Li et al., 2020; Mu et al., 2021; Rykowska et al., 2021; Silva et al., 2021a; Silva et al., 2021b; Xu et al., 2010; Zidan et al., 2021), such as dip-coating (soaking) (Guo et al., 2021; Liu et al., 2021; Maulvi et al., 2022; Wei et al., 2021) and molecular imprinting (Chu et al., 2022; Malaekeh-Nikouei et al., 2013; Raesian et al., 2021), but it is difficult to produce personalised doses using these techniques.

Printing approaches are innovative and fast-moving technologies which allow users to create customised shapes and designs (Daly et al., 2015). Drop On Demand inkjet printing is a form of two-dimensional (2D) printing in which ink droplets are deposited from a printer cartridge (Lohse, 2022). Numerous personalised drug-loaded dosage forms have been made with inkjet printing (Alomari et al., 2018; Azizi Machekposhti et al., 2021; Chang et al., 2021; Chou et al., 2021; Evans et al., 2021; Vuddanda et al., 2018; Zhang et al., 2021), including mucoadhesive buccal films (Kiefer et al., 2021) and direct printing onto the nail for onychomycosis treatment (Pollard et al., 2022). Inkjet printing onto contact lenses for glaucoma treatment would avoid the need for users to remove contact lenses for treatment, allow for personalised dosing and make point-of-care dispensing possible.

While inkjet printed anti-fungal drug-loaded contact lenses have been reported (Tetyczka et al., 2022), a means of verifying the drug load of these contact lenses have yet to be developed. To facilitate point-of-care dispensing, a non-destructive quality control method to accurately measure the amount of drug dispensed in situ is necessary. Process analytical technology (PAT) tools can perform quantitative and non-destructive analysis in real time, and have been identified in the pharmaceutical field to quantify the drug of interest. Near infrared (NIR) spectroscopy is a promising PAT tool for on-site and on-demand quantification of active pharmaceutical ingredients as it is non-destructive, rapid, specific, and requires no sample preparation (Edinger et al., 2019; Stranzinger et al., 2021; Trenfield et al., 2022; Trenfield et al., 2020; Vakili et al., 2017).

The aim of this study was to investigate the printing of a drug directly onto contact lenses and non-destructively quantify the drug load, with timolol maleate used as the model drug. The drug was printed onto both sides of the contact lens, as the chosen side may affect the drug release. The drug loading was measured, and printing multiple times was tested as a method to increase the drug dose. Measurements were made to quantify the light transmission through the contact lens following printing. A novel in-vitro dissolution apparatus was used to quantify the drug release from the contact lenses. This was also the first study to use NIR spectroscopy as a quality control measure to non-destructively quantify the drug loading of inkjet printed contact lenses.

## 2 Materials and methods

### 2.1 Materials

Timolol maleate (MW 432.49 g/moL, a Biopharmaceutics Classification System (BCS) Class I drug (Yang et al., 2007), logP 1.8 (Wishart et al., 2006), pKa 9.21 (Information, 2022)), dimethyl sulfoxide (DMSO, ≥99.9% ACS reagent grade), methanol (≥99.8% puriss ACS reagent grade), hydrochloric acid (37%), phosphate buffered saline (PBS, pH 7.4, sterile filtered) and sodium azide (reagentPlus, ≥99.5%) were purchased from Sigma Aldrich (Dorset, UK). Ultrapure grade (Type I) water (Triple Red Water Purification System, Avidity Science, Long Crendon, UK) was used. The red colourant used was Kroma Kolors Red (Kopykake Enterprise Inc., CA, USA). The contact lenses used in this study were right 1 Day Acuvue Moist Daily Disposable contact lenses (Johnson and Johnson, NY, USA) with a base curve of 8.5 mm, diameter of 14.2 mm, and power of −5.00.

### 2.2 Preparation of timolol maleate solution ‘inks’

Timolol was selected as the model drug as it is the most popular β-blocker and the reference method against which many of the marketed ophthalmic drugs have been compared with (European Glaucoma Society, 2021). To prepare a solution of timolol-loaded ink (11.2 mg/mL), timolol maleate (56.0 mg) was added to a volumetric flask (5.0 mL) with DMSO (3.50 mL). The mixture was vortexed to dissolve the drug, followed by the addition of water (1.5 mL) to bring the solution up to the 5.0 mL mark, giving a final DMSO:water solution ratio of 7:3. The solution was then stirred (30 min) and stored in the fridge (4 °C, up to 14 days). Two drops of colourant were added when required for the printed area to be seen, and the mixture was vortexed (30 s) before storage.

### 2.3 Characterisation of the commercial and in-house prepared drug inks

Various techniques described below were used to characterise the different inks and printer nozzle to ensure that the inks were printable. All measurements were conducted in triplicate. Measurements were conducted at room temperature, and at 4 °C to replicate the properties of the ink during printing.

#### 2.3.1 Density

The density of the commercial and prepared drug-loaded ink formulations was measured by placing the sample straight from the fridge (4 °C) onto on a Precisa 180A weighing balance (Precisa Balances Ltd., Livingston, UK), followed by the removal of a set volume (1.0 mL) of solution using a PIPETMAN L Fixed F1000L Gilson Pipette (Gilson Inc., Middleton, WI, USA) and recording the change in mass on the balance. The ink density was calculated by dividing the change in mass by the solution volume removed.

#### 2.3.2 Viscosity

The dynamic viscosity measurements were carried out on the *m*-VROC® viscometer (RheoSense Inc., CA, USA), controlled by the mVROC_Control_v3.1.1_AutoTemp software (RheoSense Inc., CA, USA). The temperature of the instrument was set to 4 °C using the ThermoCube cooling system (Solid State Cooling Systems, NY, USA) to mimic the printing conditions used. A glass syringe (50 μL) was filled with the filtered sample (0.22 μm filter) and subjected to a shear rate ramp of 179 to 2148 s^−1^, based on the preliminary viscosity test to determine the shear rate range. The average value was taken as the sample viscosity.

#### 2.3.3 Surface tension

Surface tension of the inks was determined using a Kibron Delta-8 microtensiometer (Kibron Inc., Helsinki, Finland) in a 96-well Dyneplate (Kibron Inc., Helsinki, Finland). A solution (50 μL, 4 °C) was added into the well, with Type 1 water used as the calibration solution throughout.

### 2.4 Calculating suitable ink properties

The aforementioned properties had to be similar to the cosmetic inks used in the commercial printer to produce drug-loaded inks that were suitable for printing. The *Z* value was calculated from Eq. (1) (Fromm, 1984):

(1) $$Z=\frac{\sqrt{d\;\rho \;\gamma }}{\mu }$$

where *d* is the diameter of the printing nozzle (μm), *ρ* is the ink density (g cm^−3^), *γ* is the surface tension (mN m^−1^), and *μ* is the dynamic viscosity (mPa s). A stable droplet at the printing nozzle is formed for *Z* between the value of 4 and 14 (Jang et al., 2009).

### 2.5 Inkjet printing process

**Fig. 1:**
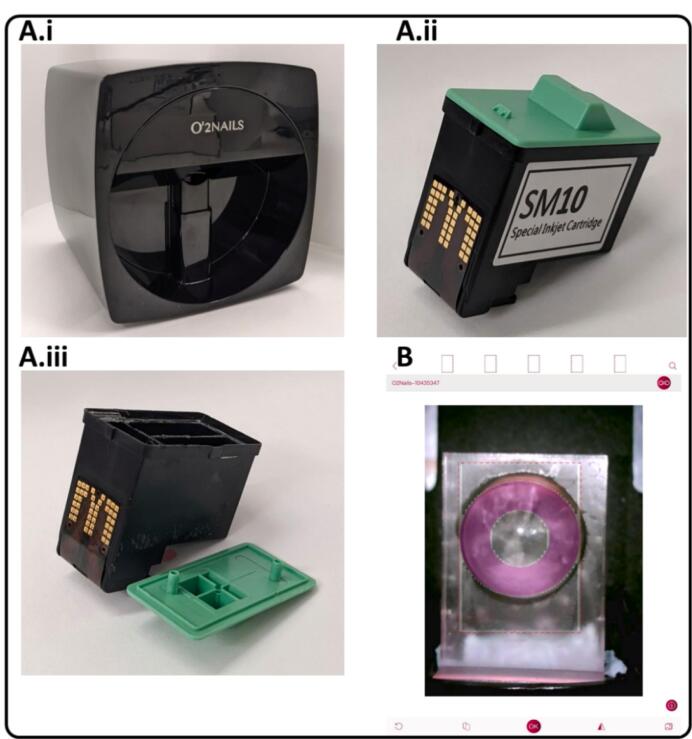
Images of the component parts used in this study. A) Photographs of the A.i) O2Nails inkjet printer, A.ii) SM10 special ink cartridge and A.iii) cleaned ink cartridge. B) Screenshot of the O2Nails app. The printer camera shows a contact lens in the lens holder with the printed shape and a magenta ring aligned to this. (For interpretation of the references to colour in this figure legend, the reader is referred to the web version of this article.)

The O2Nails V11 inkjet printer (Fig. 1A.i) (Cyber Nails, Guangzhou, China) and SM10 special inkjet cartridge (Fig. 1A.ii) (Cyber Nails, Guangzhou, China) were used for printing. This specific printer was chosen as it contains a camera capable of visualising the positioning of the object to be printed, as well as allowing the user to align the contact lens in place before and during printing. The ink cartridge contains three separate compartments for the yellow, magenta, and cyan inks. The composition of these inks is not known as it is proprietary. Control of the printer was done using the O2Nails app (Guangzhou Taiji Electronic Co, Guangzhou, China) (Fig. 1B) via the printer's WiFi. The app allows users to upload their own designs and images for printing as well as align the printed shape, which was controlled using an iPad Mini 2 smart tablet (Apple Inc., CA, USA) operating with iOS 12.4.5 software. Cleaning of the ink cartridge was conducted by first removing the external cover and sponges, filling the compartments with ethanol and ultrasonicating. The ultrasonication was carried out for 1 h at a time with the cartridge on top of a beaker to avoid any water damage. This was repeated until the ethanol remained colourless, indicating the absence of ink residue. An example of a cleaned cartridge is shown in Fig. 1A.iii.

The contact lenses had to remain wet to prevent them from drying and changing shape. Printing was conducted at cold temperatures (4 °C) to reduce the evaporation rate of water from the contact lenses. The equipment and chemicals were kept in the refrigerated environment (4 °C) between experiments. The drug-loaded ink was added into one of the compartments in the inkjet cartridge to print on the contact lens. The cartridge was then covered with parafilm and the original green lid and left to stand (30 min). The parafilm was included to help prevent spillages. The original green lid was included to trigger the cartridge detection switch inside the printer. The printing was controlled by the user using the connected mobile tablet. The nozzle of the cartridge was wiped with an ethanol-damped paper towel before printing, and 10 rectangles were printed with the colour corresponding to the compartment with the drug-loaded ink in it to purge it.

**Fig. 2:**
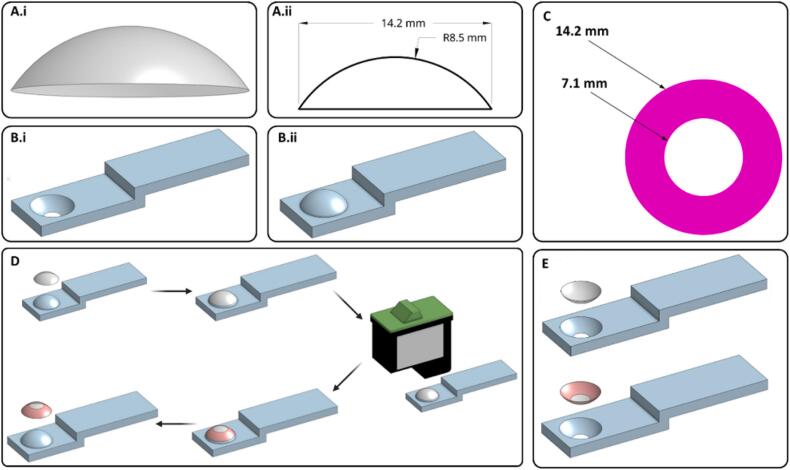
A.i) Model of the contact lens used in this study and A.ii) Measurements for the contact lenses, made using OnShape. B) Designs of the two different contact lens holders for printing on the B.i) inside face and B.ii) outside face. C) Measurements and colour of the printed ring. D) Diagram of the printing process steps with the holder for printing on the outside face, created with Biorender.com. E) Equivalent start and end images for printing on the inside face of the contact lens.

The contact lens and its dimensions are shown in Fig. 2A. An in-house holder (Fig. 2B.i and 2B.ii) was designed using OnShape (PTC Inc., MA, USA) and 3D printed using the Form 1+ 3D printer with v4 clear resin (Formlabs inc., MA, USA). These holders were used to hold the contact lens in place so that the inside or outside face of the contact lens could be printed onto with drug loaded ink. The choice of inside or outside face was anticipated to affect the drug release, and thus bioavailability of the drug. By using either method, the drug release could be tailored to the patient. The shape printed onto the contact lens was a ring with an inner diameter of 7.1 mm and an outer diameter of 14.2 mm, equal to the diameter of the contact lens (Fig. 2C). Alignment of the ring with the contact lens was manually adjusted by the user. The inkjet cartridge nozzle was wiped after every other pass to remove any excess ink and to avoid nozzle clogging. The printing process is demonstrated in Fig. 2D and E.

### 2.6 High performance liquid chromatography-ultraviolet (HPLC-UV) analysis

The concentration of timolol maleate in the liquid ink was determined using HPLC-UV, equipped with a Hewlett Packard 1260 Series HPLC system (Agilent Technologies, Cheadle, UK). The stationary phase was an Eclipse Plus C18 Column 5 μm, 150 × 4.6 μm (Agilent Technologies, Cheadle, UK), and the mobile phase was a combination of 0.01 M ammonium acetate (pH 5.0) and methanol at a ratio of 60:40 *v/v*. The aqueous solution was prepared by adding ammonium acetate (0.7708 g) to Type 1 water (1.0 L) and adjusting the pH with concentrated hydrochloric acid. The flow rate was set to 1.0 mL/min, with a column temperature of 40 °C, an injection volume of 50 μL and a UV-wavelength of 295 nm. The elution time of timolol maleate was approximately 3.4 min. A calibration curve for timolol maleate in solution was prepared between 0.4 and 400 μg/mL (R^2^ = 0.99999). The solutions were stored in sealed 1.5 mL amber glass vials (Fisher Scientific, Loughborough, UK), and 0.1 mL 5 × 31 mm glass inserts (Macherey-Nagel GmbH & Co KG, Düren, Germany) were used for samples of <1.0 mL.

### 2.7 Contact lens drug loading

Drug loading was measured by printing 3, 5, 7, and 10 passes onto the outside face of contact lenses in triplicate. Drug was only printed onto the outside face of the contact lenses as the choice of face is not expected to impact the drug load. The contact lens was stirred for 24 h in 2.0 mL PBS to release all the drug, then filtered through a 0.45 μm filter (Millipore Ltd., County Cork, Ireland) and analysed via HPLC. All results are presented as the mean ± standard deviation.

### 2.8 Light transmittance

The light transmittance (%) of unmodified, 10-pass printed drug-loaded and 10-pass printed drug free contact lenses were measured using a UV–Vis spectrophotometer (Agilent Cary 60 UV–Vis, CA, USA). All printing was onto the outside face, as the choice of face is not expected to influence the amount of light transmitted. The instrument baseline was measured before the transmittance spectra. Transmittance measurements were taken from 200 to 800 nm. The lenses were placed on a solid holder between the lamp and the detector, with the concave surface of the lens aligned perpendicular to the light beam (Conde Penedo et al., 2021).

### 2.9 Near infrared (NIR) spectroscopy

NIR reflectance spectra were measured using a MicroNIR 1700ES NIR spectrometer (VIAVI, Hertfordshire, UK) equipped with 2 vacuum tungsten lamps and an InGaAs photodiode array detector for wavelengths between 950 and 1650 nm (10,526–6060 cm^−1^). Spectra were collected using a probe with a 16 mm diameter collection optic attached to the MicroNIR device. Contact lenses were placed between the probe and a sapphire window accessory with an anti-reflection coating. A 99% spectralon reference standard (VIAVI, Hertfordshire, UK) was used for the acquisition of dark and reference spectra for instrument calibration prior to spectra acquisition.

Contact lenses were printed with 3, 5, 7, and 10 passes of timolol maleate (11.2 mg/mL) on the outside face in triplicates. The choice of face was not expected to affect the NIR measurement. Each contact lens was analysed at three different points with the probe pointed at the outside face. The final spectrum for each contact lens was the average of the spectra recorded over the three points. Data was acquired using VIAVI MicroNIR Pro software (VIAVI, Hertfordshire, UK). Data pre-processing, multivariate data analysis, and modelling was performed with a separate python 3.10 script. The model was trained using a train:test split of 80:20 to measure the performance of the model in a real scenario on unseen data. Partial least squares (PLS) regression was performed on the datasets, with 10-fold cross validation with 3 repeats, to build calibration models. PLS model graph of NIR predicted vs. HPLC determined timolol content was created using GraphPad Prism 8 (San Diego, California, US). Following NIR analysis, each individual contact lens was quantitatively analysed for drug content via HPLC following the methodology described in Section 2.7.

### 2.10 In vitro dissolution test

**Fig. 3:**
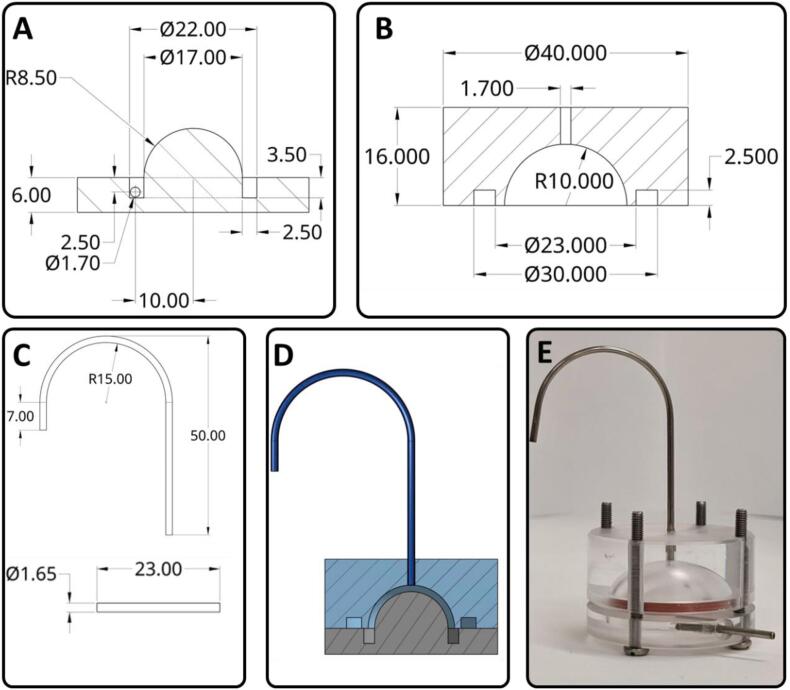
Schematic of the dissolution vessel. A) Bottom part of rig. B) Top part of rig. C) Metal piping parts. D) Full assembly. E) Photograph of the constructed vessel.

Contact lenses were printed with 10 passes of timolol maleate (11.2 mg/mL) on the inner and outer face in triplicate. Release studies were conducted in an in-house flow rig model (Fig. 3). The dimensions of the sample chamber had an outer diameter of 20 mm, an inner diameter of 17 mm, and a capacity of 2220 ± 240 μL. The rigs were rinsed, cleaned, and dried prior to each experiment. Drug-loaded contact lenses were gently placed in each rig and the models were assembled, filled with buffer (PBS, pH 7.4 with 0.05% sodium azide) and placed on a heating plate at 37 °C. The models were connected to an 8-channel Ismatec peristaltic pump (Michael Smith Engineering Ltd., Woking, UK) with a 2.0 μL/min flow rate at 37 °C, which is similar to human ciliary body inflow (Abu-Hassan et al., 2014). Samples were collected (1, 2, 3, 4, 5, 6, 8, 10, 18, and 24 h) using glass vials via the outflow port, which were replaced at each sampling point.

The volume of the model was measured by weighing an empty vessel with one end blocked, filling the vessel with water, and re-weighing. The volume of the model was calculated by dividing the weight by 0.97713 g/cm^3^, the density of water at 25 °C.

## 3 Results and discussion

### 3.1 Ink selection and characterisation

The ink solution composition was selected to give high drug loads while remaining printable. The physicochemical properties of DMSO-water combinations have previously been reported (LeBel and Goring, 1962; Markarian and Terzyan, 2007). Timolol maleate is more soluble in DMSO than in water (16 vs. 0.2 mg/mL, respectively) (Chemical, 2022) however, high DMSO proportions have been reported to cause ocular inflammation (> 70%) (Hanna et al., 1977), therefore, studies were conducted with a 7:3 DMSO:water ratio. A timolol maleate concentration of 11.2 mg/mL was chosen as this was close to its solubility limits. A small amount of liquid red colourant was added so the printed area could be visualised.

Density, surface tension and viscosity measurements of the commercial ink, DMSO:water mixture at room temperature (∼25 °C) and 4 °C, and timolol maleate loaded DMSO:water were recorded (Table 1). Previous data indicates the nozzle diameter was 21.0 ± 2.2 μm, and that the commercial inks have an average density of 1.03 g/cm^3^, viscosity of 2.17 mPa.s, and surface tension of 35.61 mN/m, giving a *Z*-value of 12.8 (Pollard et al., 2022). All the solutions showed very similar densities and reasonably similar surface tensions. The surface tension was higher for the DMSO:water combinations, but this did not change with cooling nor with the addition of timolol maleate and colourant. However, the viscosity of the solutions at 4 °C was much higher than that of the commercial inks and of the solutions at room temperature. Cooling significantly impacted the viscosity of the solution and, in turn, caused the Z-value to be much lower. The viscosity was unchanged with the addition of timolol maleate and colourant, indicating that these additions did not have a significant impact on the physicochemical properties of the solution. Lower proportions of DMSO may better mimic the commercial inks, however, these would reduce the drug loading, and so were not used.

**Table 1:** Solution characterisation of density, viscosity, and surface tension, and the Z value for a nozzle diameter of 21.0 ± 2.2 μm.

Solution	Density (g/cm^3^)	Viscosity (mPa.s)	Surface tension (mN/m)	Z value (d = 21.0 μm)
Commercial ink at 25 °C	1.03 ± 0.02	2.17 ± 0.13	35.61 ± 0.08	12.8 ± 0.9
70:30 DMSO: H_2_O at 25 °C	1.100 ± 0.004	4.321 ± 0.004	52.5 ± 0.4	8.0 ± 0.4
70:30 DMSO: H_2_O at 4 °C	1.094 ± 0.006	8.890 ± 0.006	53.77 ± 0.17	3.95 ± 0.21
11.2 mg/mL timolol solution at 4 °C	1.101 ± 0.012	9.086 ± 0.009	52.57 ± 0.74	3.83 ± 0.20

### 3.2 Inkjet printing onto contact lenses

**Fig. 4:**
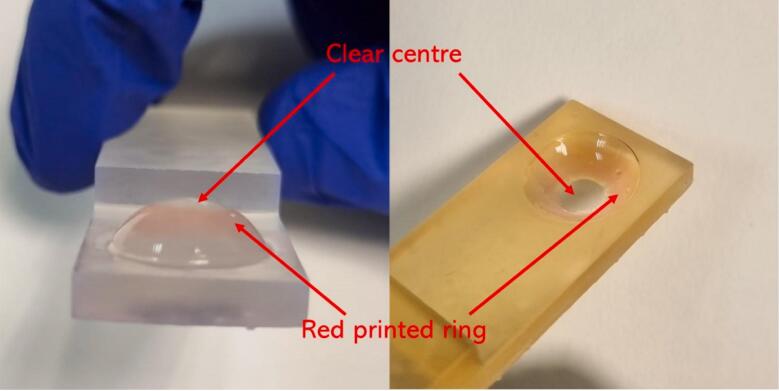
Different angles of the printed contact lens.

Timolol maleate loaded ink (with or without the colourant) was successfully printed onto both sides of the contact lenses (Fig. 4). The ring shape was chosen for printing to avoid obstruction of vision. The drug-loaded ink was tested for printing at both room temperature and 4 °C. The printer was able to print with the ink and reproduce the desired shapes at both temperatures. The Z-value of the ink at 4 °C was below the literature ideal printing range of 4–14 (Jang et al., 2009), which is expected to give a lower positional accuracy and printing resolution. However, the inks were very close to the ideal printable range since the Z-values were close to 4. From observation, the accuracy of the printing did not appear to be substantially affected. The printer was able to print onto both the inside and outside of the contact lens. This has the potential to alter the drug release rate and bioavailability in vivo. If the drug is printed on the outside face of the contact lens, the contact lens will act as a barrier between the drug and the eye surface, limiting absorption. Additionally, the movement of the eyelids may speed up the release of the drug from the contact lenses. When printing on the inside face of the contact lens, the drug would be in direct contact with the eye, which may increase absorption. The drug release here would not be affected by the movement of the eyelids.

### 3.3 Characterisation of drug-loaded contact lenses

**Fig. 5:**
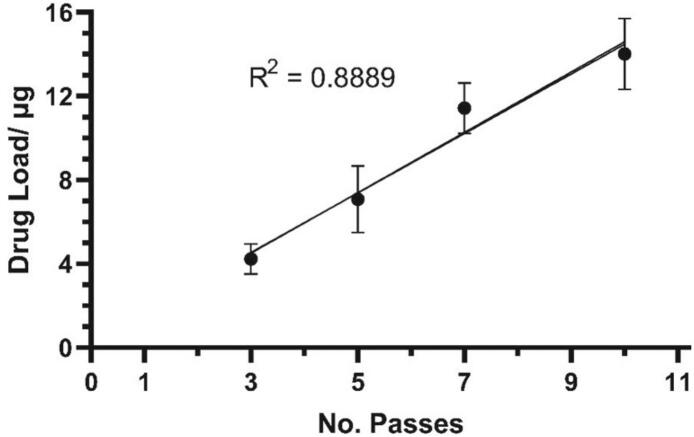
Plot of drug loading against number of passes.

The results from measuring drug load with different numbers of prints onto contact lenses are shown in Fig. 5. The drug load appears to increase linearly (R^2^ = 0.8889) with the number of passes as expected. The results also demonstrate that timolol maleate was both printable onto a contact lens and extractable. It was decided to limit the maximum number of passes to 10 due to the time taken to print high numbers of passes. This could be printed in 15 min per contact lens.

**Fig. 6:**
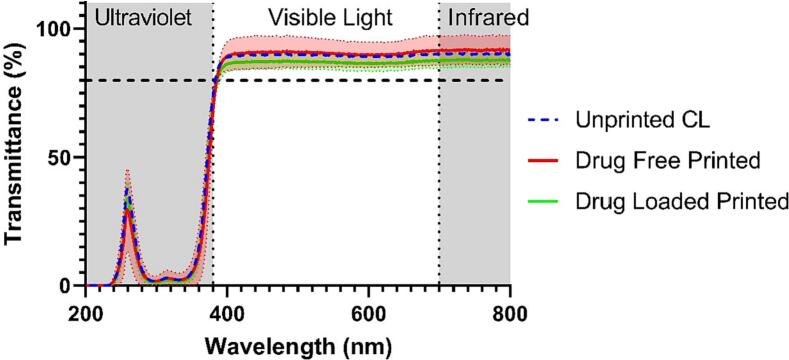
Light transmission of the drug-loaded and non-drug loaded CLs with 10 passes, and the unmodified commercial contact lenses. The horizontal dashed line indicates 85% transmittance, while the dark grey regions indicate the ultraviolet and infrared spectrum. The coloured shaded regions represent the standard deviation in the measurement.

Light transmittance was measured through both drug-loaded and drug-free printed contact lenses at 10 passes, and the commercial contact lenses without printing. The inks used here did not contain red colourant as this would absorb light. Light transmittance of all contact lenses showed values above 85% in the visible range (380 to 700 nm). No significant differences in light transmittance were observed between the printed drug loaded CLs and drug free CLs (Fig. 6). The presence of a UV blocking filter in the lenses significantly reduced the transmission of UV radiation below 380 nm (Lira et al., 2009). The high transmittance of the drug-loaded contact lenses in the visible region indicated that the drug-loaded contact lenses would not interfere with normal vision, thus making them suitable for use.

### 3.4 Quality control with near infrared (NIR) spectroscopy

**Fig. 7:**
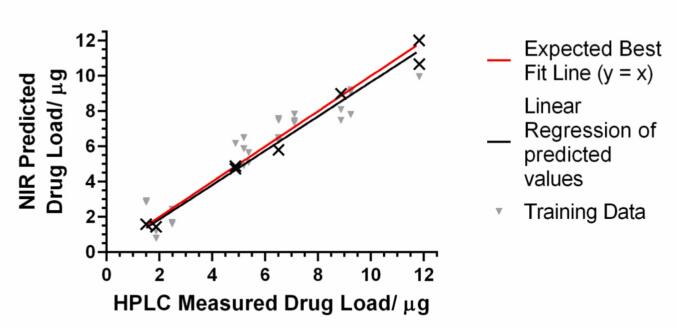
PLS model of NIR predicted vs. HPLC determined timolol content. The expected best fit line is for the actual concentration equal to the predicted concentration.

To create a multivariable calibration model, different spectral pre-processing techniques were evaluated. Data pre-treatment was required to eliminate and minimise variability, extract relevant chemical information and improve the accuracy of quantification (Rinnan et al., 2009). In this study, several PLS models were developed with three different pre-treatment filters and their combinations (Standard Normal Variate, Savitzky-Golay smoothing, and Multiplicative Scatter Correction) applied to the spectra. The model with the lowest root mean square error (RMSE) value and higher linearity (largest R^2^) was selected. The selected model used wavelengths between 950 and 1650 nm and a 2nd derivative (Savitzky–Golay with a filter width of 25 and a 2nd polynomial) pre-processing technique. The correlation between NIR predicted values and the reference concentrations determined with HPLC is shown in Fig. 7. The model showed a good linearity (R^2^ = 0.9120) with an RMSE of 1.1196 for the total of 12 samples over a timolol maleate mass range from 1.50 to 11.83 μg (3, 5, 7 and 10 passes), confirming that the NIR results were proportional to timolol maleate concentration in the contact lenses in the stated range. Hence, NIR provides an accurate method for quality control via non-destructive drug load measurements.

### 3.5 In vitro drug release study

**Fig. 8:**
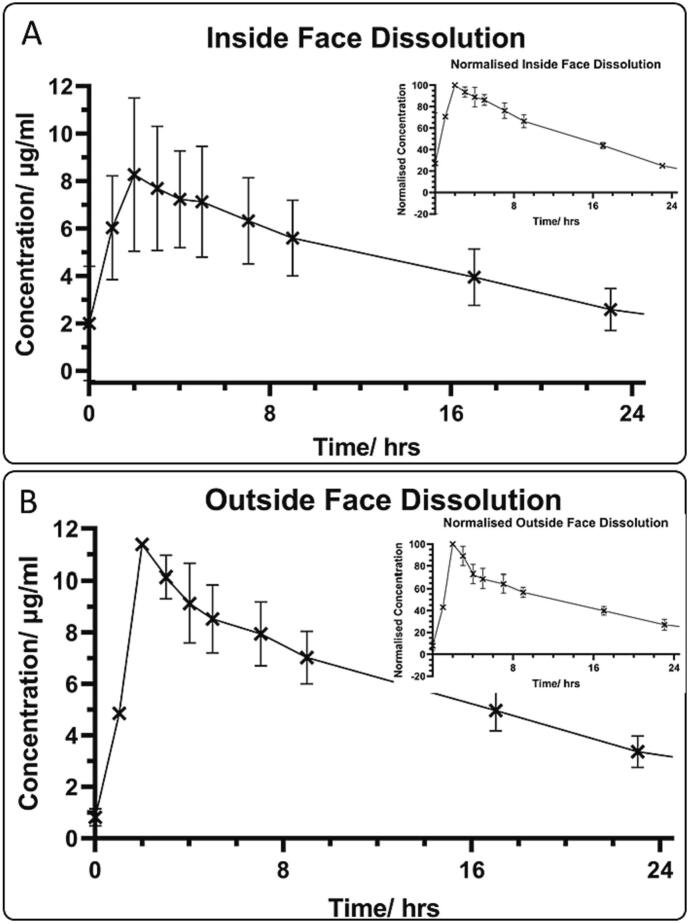
Results from the in vitro drug release study. Error bars are ±1 standard deviation. A) Measurements of concentration collected over time with the drug printed on the inside face of the contact lens. Insert – plot with the concentration relative to the maximum concentration for the inside face dissolution. B) Measurements of drug concentration over time for drug printed on the outside face of the contact lens.

Contact lenses were loaded with 10 passes of drug on the inside (Fig. 8A) or outside (Fig. 8B) face and the drug release measured using an in-house designed rig, which was designed to mimic the behaviour of contact lenses in vivo. The 2.0 μL/min is similar to the aqueous humour inflow in the human eye (Goel et al., 2010; Radenbaugh et al., 2006). The curved nature of the vessel allowed the contact lens to retain its normal shape.

One consideration was the volume of the rig. The volume used was much higher than previous studies (Angkawinitwong et al., 2017; Xu et al., 2021); 2.2 mL vs 200 μL, respectively. However, these models are too small for the contact lenses to fit and retain their shape as they were predominantly designed for subconjunctival formulations. The curved bottom part of the rig meant only one surface of the contact lens was in contact with the liquid, which is more realistic.

Two key factors were necessary in the design of the rig model. Firstly, the delay time from the outlet piping was considered. Using a similar method used for measuring the volume of the rig model, the volume of the outlet pipe was measured as 121 ± 7 μL, equivalent to a time delay of 1.00 ± 0.06 h. Additionally, the continuous pumping may lead to continual dilution of the drug sample, which needs to be correctly identified as the dilution of the existing drug and not as continued drug release. Once all the drug was released, the decay would be exponential with a characteristic time of $\tau =\frac{V}{r}$, where *τ* was the characteristic decay time, *r* was the rate of infusion, and *V* was the volume of the vessel (Supplementary Material 1).

The drug concentration from the in vitro release model showed a peak in the C_max_ (11.38 ± 0.19 and 8 ± 3 μg/mL for the outside and inside face respectively) at 2 h (Fig. 8), followed by a gradual decrease in concentration. The lower maximum concentration for the inside face compared to the outside face can be attributed to the drug being between the contact lens and the curved part of the dissolution apparatus for inside face printing. Hence, the drug either has to diffuse through the contact lens or the solution has to penetrate between the contact lens and the curved surface. These are both a greater barrier to drug being freely in the solution compared to the drug being on the outside face, and thus in constant contact with the bulk of the liquid. Hence, the inside face has a lower concentration maximum.

The inside face dissolution results also showed a large variation in concentration. This may be due to differences in adhesion of the contact lens to the dissolution rig giving different amounts of liquid able to pass under the contact lens. The variation may also be partly due to differences in alignment during printing giving different drug loads. Conceivably, a purpose-built inkjet printer could have a camera to accurately verify alignment of the contact lenses and reduce drug load variations.

The decay constant of the exponentially decaying parts of the curves corresponds to a vessel volume of 2410 ± 70 and 2530 ± 70 μL for the inside and outside faces respectively. The measured vessel volume was 2220 ± 240 μL. The agreement of these values suggests the drug concentration decay was indeed due to continuous dilution from the pump. It is not obvious at which point the drug is fully released and starts being diluted, but dilution appears to start at some point between 3 and 7 h. It is evident that the drug release was not instantaneous, and that the drug-loaded contact lenses released drug in the span of a few hours. This was a significant improvement over standard eye droplets, which have a pre-corneal retention time of approximately 10 min (Jumelle et al., 2020).

### 3.6 Discussion

In this work, we have demonstrated the effectiveness of inkjet printing for dispensing drugs onto contact lenses, and NIR as a non-destructive PAT tool for dose verification. Inkjet printing of drugs onto contact lenses boasts a number of advantages compared to eye drops, the current standard dosage form. Eye drops have varying sizes (Lederer Jr. and Harold, 1986; Moore et al., 2017), whereas inkjet printing onto contact lenses allows for a controlled and known dose to be dispensed. In addition, inkjet printing improves the retention time (at least 3 h compared to 10 min for eye drops) and is much more applicable for contact lens users. Around 140 million people globally currently wear contact lenses, which is set to rise as a result of increasing product availability, low-cost options, and an improvement on both the quality of life and vision without changing physical appearance (Bhargava, 2020).

Quality control is a crucial step for decentralised dispensing, as the amount of drug given must be measured non-destructively in order for the drug product to meet the necessary regulations and specifications. NIR spectroscopy is an industry standard analytical method for quality control that can help to overcome limitations in translating 3D printed pharmaceuticals into clinical settings (Seoane-Viaño et al., 2021). This technology has already proven capable of quantifying drugs in 3D-printed dosage forms (Trenfield et al., 2020), but has never been used to quantify drugs in contact lenses. Here, NIR with a 2nd derivative (Savitzky–Golay) filter showed excellent linearity between the predicted and actual drug dose. Hence, the combination of inkjet printing with NIR presents a considerable opportunity for the personalised, point-of-care loading of glaucoma therapies. The point-of-care loading would also mean that the contact lens storage would not be affected, since only small volumes are printed.

Printing was demonstrated on both the inside and outside face of the contact lens. The advantage of being able to do either is that this could potentially be used to alter the drug release. Printing on the outside is anticipated to lead to faster dissolution than inside printing due to the eyelid movement, whereas inside face printing is expected to lead to greater bioavailability; increased concentration of the drug near the cornea surface means more drug is able to diffuse through to give higher bioavailability (Maulvi et al., 2016). Indeed, contact lenses have previously been shown to have greater bioavailability and greater reductions in intraocular pressure with lower drug loads using soaked contact lenses (Hsu et al., 2015). The differences between printing on the inside and outside of the contact lenses should be studied in vivo, in future work. Care would be needed when printing on the outside to avoid smearing.

Compared to other contact lens loading methods, inkjet printing has many favourable attributes. Personalised treatment is of high clinical need in ophthalmology (Ong et al., 2013), and inkjet printing can provide this. Additionally, inkjet printing could be used to produce different doses in each eye, such as for unilateral glaucoma. It is possible for inkjet printing to manufacture the dosage forms at the point-of-care, and the drug dispensing process is much more straight-forward than direct embedding. Point-of-care production could be done at a convenient place for the patient, which is especially useful for glaucoma patients as they are less mobile (Friedman et al., 2007; Turano et al., 1999). Drug loaded contact lenses produced by soaking do not allow for users to control the dose, and shows rapid drug release (〈1*h*) (Wuchte et al., 2021). Direct embedding does allow for tailored dosing, but it also has multiple steps in the manufacturing process, such as sonication, curing and washing, which make it unsuitable for point-of-care dispensing (Maulvi et al., 2020; Maulvi et al., 2015). In comparison, we have shown that inkjet printing shows a more prolonged release than soaking, and allows for controlled dosing. This inkjet printing method also produced higher drug loads than previous inkjet printing loaded contact lenses (Tetyczka et al., 2022).

Inkjet printheads can also easily contain multiple different inks, and so inkjet printing could allow for multi-drug therapies. Additionally, diffusion barriers, such as vitamin E, could be printed to give a more controlled release profile.

Further work into inkjet printing could also help to overcome some of the limitations with the method. The method presented here has fairly low drug loads. Development of a custom-built printer could better match the properties of the ink to give bigger droplets and thus higher drug loads. Additionally, other issues from printing, such as possible recrystallization of the drug or disturbances in the optical properties of the lenses, should be thoroughly checked. Due to the drug loads used and the light transmission results, it is not expected that these issues will occur.

## 4 Conclusions

The printing of timolol maleate was demonstrated with an adapted commercial inkjet printer. The drug solutions were tailored to match the commercial inks, and the drug dosing of timolol maleate was successfully controlled by printing multiple times. NIR measurements with a Savitzky–Golay filter was successfully demonstrated as a means for quality control by measuring the drug load non-destructively. A novel in vitro release apparatus was designed to mimic the drug dissolution from a contact lens around the eye. Results from this study indicated that the contact lenses were capable of releasing drug over multiple hours, much longer than the standard eye droplet retention time. As such, this system was an efficient method for improving the drug release from the eye using printed-on contact lenses. Additional work to modify the printer would enable the drug dose to be increased, while alterations to the contact lenses could allow for more controlled drug release, thus enhancing the method's potential further. In summary, inkjet printing is a leading technology that has the potential to improve drug release from the eye for the treatment of various front of the eye ocular diseases.

## CRediT authorship contribution statement

**Thomas D. Pollard:** Data curation, Formal analysis, Investigation, Methodology, Writing – original draft, Writing – review & editing. **Iria Seoane-Viaño:** Data curation, Formal analysis, Investigation, Methodology, Writing – original draft, Writing – review & editing. **Jun Jie Ong:** Data curation, Formal analysis, Investigation, Methodology, Writing – original draft, Writing – review & editing. **Patricija Januskaite:** Data curation, Formal analysis, Investigation, Methodology, Writing – original draft, Writing – review & editing. **Sahar Awwad:** Supervision, Resources, Writing – original draft, Writing – review & editing. **Mine Orlu:** Supervision, Resources, Writing – review & editing. **Manuel F. Bande:** Conceptualization, Supervision, Writing – original draft. **Abdul W. Basit:** Conceptualization, Methodology, Project administration, Resources, Supervision, Writing – review & editing. **Alvaro Goyanes:** Conceptualization, Methodology, Project administration, Resources, Supervision, Writing – review & editing.

## Declaration of Competing Interest

The authors declare that they have no known competing financial interests or personal relationships that could have appeared to influence the work reported in this paper.

## Data Availability

Data will be made available on request.

## Appendix A Supplementary data

Derivation of the equation for drug dilution Image 1
