# Supplementary material for: Inkjet drug printing onto contact lenses: Deposition optimisation and non-invasive dose verification
Source: Int J Pharm X. 2022 Dec 20;5:100150. doi: 10.1016/j.ijpx.2022.100150 (PMC9804110; doi:10.1016/j.ijpx.2022.100150)
Supplement: Supplementary file 1 — Derivation of the equation for drug dilution [file mmc1.docx]

**SUPPLEMENTARY MATERIAL**

**Inkjet drug printing onto contact lenses: deposition optimisation and non-invasive dose verification**

Thomas Pollard^a^, Iria Seoane-Viaño^a,b^, Jun Jie Ong^a^, Patricija Januskaite^a^, Sahar Awwad^a^, Mine Orlu^a^, Manuel F. Bande^c^, Abdul W. Basit^a,d,^*, Alvaro Goyanes^a,d,e,^*

^a^ Department of Pharmaceutics, UCL School of Pharmacy, University College London, 29-39 Brunswick Square, London, WC1N 1AX, UK

^b^ Department of Pharmacology, Pharmacy and Pharmaceutical Technology, Paraquasil Group (GI-2109), Faculty of Pharmacy, and Health Research Institute of Santiago de Compostela (IDIS), University of Santiago de Compostela (USC), Santiago de Compostela, 15782, Spain.

^c^ Department of Ophthalmology, University Hospital of Santiago de Compostela, Ramon Baltar S/N, Santiago de Compostela, 15706, Spain.

^d^ FabRx Ltd., Henwood House, Henwood, Ashford TN24 8DH, UK

^e^ Departamento de Farmacología, Farmacia y Tecnología Farmacéutica, I+D Farma Group (GI-1645), Facultad de Farmacia, iMATUS and Health Research Institute of Santiago de Compostela (IDIS), Universidade de Santiago de Compostela (USC), Santiago de Compostela, 15782, Spain.

*Correspondence: A.G; a.goyanes@fabrx.co.uk, A.B.; a.basit@ucl.ac.uk

**Supplementary Material 1: Drug Dilution Derivation**

The mass of drug dissolved within the vessel can be mathematically modelled. The drug is assumed to be uniform within the solution. Solution is constantly pumped through the vessel at rate $r$. Since the drug is uniform, the concentration at time $T$, $c(T)$, can be expressed as;

$$c\left( T \right)=\frac{M\left( T \right)}{V}$$

where V is the volume of the vessel.

The drug mass can increase from more drug being released from the contact lens into the solution, with a release rate of $Q(T)$. Additionally, the drug mass can be reduced via the continuous pumping of the solution.

For drug mass $M$ at time $T$, then, at a small time later ($T+\delta T$);

$$M\left( T+\delta T \right)\approx M\left( T \right)+Q\left( T \right)\delta T-r c\left( T \right) \delta T$$

Here, $M(T)$ represents the mass of the drug before the time step, $Q\left( T \right)\delta T$ is the mass of drug added from drug release from the contact lens, and $-r c(T) \delta(T)$ is the mass of drug that is pumped out of the vessel in time $\delta(T)$.

Then, be rearranging;

$$M\left( T+\delta T \right)-M\left( T \right)=\delta M\approx Q\left( T \right)\delta T-\frac{rM}{V}\delta T$$

Where $M=M(T)$ is implied. Dividing through by $\delta T$;

$$\frac{\delta M}{\delta T}\approx Q\left( T \right)-\frac{r}{V}M$$

Taking the limit of this as $\delta T\to0$ gives a definite expression;

$$\frac{dM}{dT}=Q\left( T \right)-\frac{r}{V}M$$

Dividing by $M$ gives;

$$\frac{1}{M}\frac{dM}{dT}=Q\left( T \right)-\frac{r}{V}$$

The exact form of $Q(T)$is not known. If the point at which all the drug is released from the contact lens occurs is set to $T=0$, then from this point onwards; $Q\left( T\geq0 \right)=0$. Here, the total drug mass in the solution is $M\left( T=0 \right)=m_{0}$ and hence, by rearranging the expression and integrating;

$$\int_{M=m_{0}}^{m} \frac{1}{M}dM=\int_{T=0}^{t} -\frac{r}{V}dT$$

This integrates to give;

$$\ln\left( m \right)-\ln\left( m_{0} \right)=-\frac{r}{V}t$$

This can be rearranged to give;

$$m\left( t \right)=m_{0} e^{-\frac{r}{V}t}$$

By setting $\tau=\frac{V}{r}$, then

|  | $m\left( t \right)=m_{0}e^{-\frac{t}{\tau}}$ | (1) |
| --- | --- | --- |

This expression is exponential decay with a time constant of $\tau$.

In this experiment, the mass of drug isn’t measured, but the concentration collected between timepoints. The concentration can be found by dividing by $V$ to give;

$$c\left( t \right)=\frac{m_{0}}{V}e^{-\frac{t}{\tau}}$$

To a first approximation, the average concentration (concentration measured) can be regarded as the concentration midway between measurement points. Then, by using logarithms;

|  | $\ln\left( c\left( t \right) \right)=\ln\left( \frac{m_{0}}{V} \right)-\frac{t}{\tau}$ | (2) |
| --- | --- | --- |

Plotting this should give a linear graph, with gradient $-\frac{1}{\tau}=-\frac{r}{V}$.
